# Clinical Trials in Alzheimer's Disease: A Hurdle in the Path of Remedy

**DOI:** 10.1155/2020/5380346

**Published:** 2020-04-01

**Authors:** Alexandra E. Oxford, Erica S. Stewart, Troy T. Rohn

**Affiliations:** Department of Biological Sciences, Boise State University, Science Building, Room 228, Boise, Idaho 83725, USA

## Abstract

Human clinical trials seek to ameliorate the disease states and symptomatic progression of illnesses that, as of yet, are largely untreatable according to clinical standards. Ideally, clinical trials test “disease-modifying drugs,” i.e., therapeutic agents that specifically modify pathological features or molecular bases of the disease and would presumably have a large impact on disease progression. In the case of Alzheimer's disease (AD), however, this approach appears to have stalled progress in the successful development of clinically useful therapies. For the last 25 years, clinical trials involving AD have centered on beta-amyloid (A*β*) and the A*β* hypothesis of AD progression and pathology. According to this hypothesis, the progression of AD begins following an accumulation of A*β* peptide, leading to eventual synapse loss and neuronal cell death: the true overriding pathological feature of AD. Clinical trials arising from the A*β* hypothesis target causal steps in the pathway in order to reduce the formation of A*β* or enhance clearance, and though agents have been successful in this aim, they remain unsuccessful in rescuing cognitive function or slowing cognitive decline. As such, further use of resources in the development of treatment options for AD that target A*β*, its precursors, or its products should be reevaluated. The purpose of this review was to give an overview of how human clinical trials are conducted in the USA and to assess the results of recent failed trials involving AD, the majority of which were based on the A*β* hypothesis. Based on these current findings, it is suggested that lowering A*β* is an unproven strategy, and it may be time to refocus on other targets for the treatment of this disease including pathological forms of tau.

## 1. The Challenges of Treating CNS Disorders

Before discussing recent clinical trials involving Alzheimer's disease (AD), it is important to highlight some of the challenges associated with treating CNS disorders. The challenges specific to the CNS derive, at least in part, from the fact that the scientific understanding of the brain is not as advanced as some other physiological systems. In the majority of disorders within the CNS, there remains minimal or, in some cases, no understanding as to the pathophysiology of the condition of interest. Thus, designing therapeutics whereby molecular targets are not well defined is an obvious hurdle that must be overcome. This has led to the cold reality that despite the high and growing prevalence as well as the substantial economic burden, there are no disease-modifying therapies for many neurological disorders and neurodegenerative diseases. In addition, compared to other systemic diseases, failure rates in late stage clinical trials are high for neurologic and psychiatric diseases due in part to the complexity of the human brain [1]. This makes the development process longer compared to other therapeutic areas and is related possibly to greater safety risks for volunteers and patients, particularly at early phases of clinical trials. Another confounding subjective factor is the placebo effect, in which a patient's expectation of therapeutic benefit can interfere with the response to drug administration [2]. Indeed, the placebo effect pervades all clinical trials, particularly CNS trials [3]. For example, the failure of many recent neurological clinical trials, such as those for pain, Parkinson's disease, and schizophrenia, is a direct result of high placebo effects [3].

A substantial proportion of CNS drug trials also rely on subjective endpoints, which can increase the potential for variability in the data. For example, many AD trials rely on endpoints such as subjective memory improvement or the use of the Alzheimer's Disease Assessment Scale-cognitive (ADAS-cog) to capture changes in cognition as the primary measures of efficacy. Clinicians and trial sites vary widely in their experience in administrating assessment instruments, leading to unintentional variability of data. In addition, clinical assessments using scales to measure cognitive impairment, disability, quality of life, or global disease severity are affected by symptomatic effects of therapies and, in the short term, cannot differentiate this effect from disease modification. These factors combine to make it nearly impossible to demonstrate proof of efficacy in phase II-III trials [4].

In addition to these subjective endpoints, there are other barriers that have limited the success of developing therapeutics for CNS disorders. Unique challenges start at the preclinical stage due to the complexity of the human brain and the limited possibility to study drug candidates in the relevant tissue environment. More importantly, the aspect of the blood-brain barrier (BBB) creates multiple issues in drug penetration. Therapeutic drugs that show early promise during drug development often fail to successfully clear subsequent clinical trials due in part to their inability to cross the BBB. It has been estimated that more than 98% of small molecule drugs and nearly 100% of large molecule drugs are precluded from drug delivery to the brain as a result of the BBB [5].

**Figure 1:**
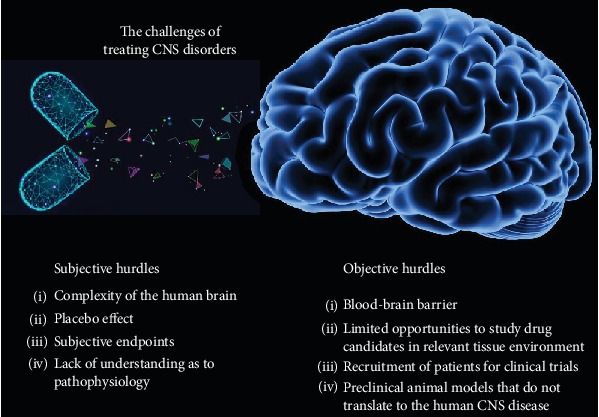
The challenges of treating CNS disorders. Both subjective and objective hurdles must be surpassed when developing pharmaceuticals for the treatment of CNS disorders and diseases. Overcoming these hurdles has proven difficult leading to a minimum an increase in time and cost for drug development and at a worse to many drug failures that would otherwise be realized for a systemic-related disorder.

Probably one of the most surprising objective hurdles that must be overcome in the treatment of CNS disorders is the recruitment of patients. Recruitment is the slowest and most expensive aspect of clinical trials and represents a major barrier to clinical research progress. The challenge of patient recruitment is due, at least in part, to poor public awareness about many CNS diseases, which may result in delayed diagnosis. In AD, this has been a long-standing problem [6–8] for reasons including that only approximately 20-25% of people are eligible to participate in AD trials [9] and that trials are seeking participants who are either in prodromal or asymptomatic phases of the disease [10].  Figure 1 summarizes the challenges associated with developing therapeutic agents for the treatment of CNS disorders and diseases.

## 2. Brief Overview of FDA Clinical Trials

In the United States, clinical trials are regulated by the Food and Drug Administration (FDA) to ensure that drugs reaching the market are both efficacious in the treatment of the intended disorder or illness and safe for the intended patient population [11]. Having a molecular entity approved for the treatment of a disease or disorder is no small feat: the process for new drugs is divided into 2 sections (preclinical and clinical) and typically spans 12-15 years at a cost of $2.6 billion [12, 13]. Approximately 5 of 5,000 new drugs complete the preclinical phase and advance to clinical trials in humans. Of those five, one drug is typically approved by the FDA and reaches the marketplace [14].

Clinical trials consist of four distinct phases identified as I-IV, as well as a preclinical phase, and a phase 0 trial representing an exploratory trial [13, 15] (Table 1). In its entirety, the clinical trial process may take anywhere from 12-15 years and, with 80% of clinical trials failing after phase I or II, offers no guarantee of success [13].

Though much of the data obtained at each step of the clinical trial process is available to the public through the NIH clinical trials database (clinicaltrials.gov), this is still a relatively new level of transparency, having come into place in 2007. Now, over ten years later, it is important to note that regulation and enforcement of timely reporting remain somewhat lack luster, and therefore, not all clinical trial information may be publicly available. This is still the case even after a 2017 collaboration by the NIH and FDA to litigate the so-called final rule meant to clarify and allow for the enforcement of this such reporting [16].

### 2.1. Preclinical Phase

Clinical trials begin with preclinical testing that typically lasts 6.5 years. The preclinical phase aims at gathering information on the pharmacokinetics, chemistry, manufacturing plan, and potential quality control of the proposed drug [15]. At the preclinical stage, risk is determined by conducting toxicity studies using *in vitro* and *in vivo* models. For *in vivo* studies, at least two different mammalian species are required (commonly mice and rats) [11]. The use of mice as a model system in preclinical testing may be a contributing factor to the high failure rate of AD trials, as the findings derived from these studies do not always translate to human neurophysiology [17].

### 2.2. Phase 0

Because only about 10% of IND applications result in clinically approved drugs [18] and because drug development is a lengthy, expensive, and risky proposition, in some cases, early testing may be performed to assess whether a drug engages with its expected target and is therefore more likely to have the anticipated clinical effect in human subjects [19]. The main goal of phase 0 trials is to acquire, in a relatively small group of subjects receiving nontoxic doses of the drug, information that would aid in the design and potential success of subsequent larger phase I-II trials (Table 1). Another potential goal of a phase 0 trial is to determine whether a mechanism of action defined in nonclinical models can be achieved in humans [20]. Phase 0 can eliminate drugs that do not measure up to even the most rudimentary requirements and thus can save time and money [21].

### 2.3. Phases I-IV and NDA

Following the approval of an IND by the FDA or successful completion of a phase 0 trial, clinical trials may begin in phase I. Unless a phase 0 trial has been carried out, phase I clinical trials represent the first incidence of human exposure to the drug candidate and therefore must focus their efforts on testing in a small sample of healthy volunteers, avoiding, for the time, adverse effects that may be unique to, or more extreme in, a diseased population [11]. Phase I studies are primarily safety trials and are interested in further elucidating the toxicity and pharmacokinetic factors associated with the treatment [22]. These are typically single-blind studies that involve a small number of subjects (Table 1) [13].

Phase II trials are controlled clinical studies evaluating the effectiveness of the drug in a specific disease and defining an effective dose that provides the optimal benefit-risk profile for the use of the drug [13]. In phase II, studies are conducted at a larger scale (dozens to hundreds) on patients with the disease that the drug is intended to treat. In addition to determining side effects, phase II studies begin to assess efficacy [13]. The average duration is 2 years, and after phase II, the FDA and drug sponsors collaborate on phase III study designs [22]. It is noteworthy that 80% of all drugs tested are abandoned by their sponsors after phase I or II because of excessive toxicity or lack of efficacy [23].

Phases III and IV differ from the previously discussed trial designs due largely to their magnitude and are the final confirmation of safety and efficacy. Phase III studies are large controlled trials involving thousands of people in the target disease population. The trials evaluate effectiveness, monitor side effects, and compare the drug with commonly used alternative therapies [15]. These studies continue to evaluate the safety and efficacy of the proposed treatment, but at a large enough scale to ensure that any observed results are statistically significant [11] [23]. A key aspect of phase III trials is that they must include enough patients to have at least an 80% chance of finding a clinical effect if it really exists. This is often referred to as the power of the study [23].

Upon the completion of phase III studies, a New Drug Application (NDA) is submitted to the FDA [11]. This interim period is sometimes referred to as the FDA approval phase and typically lasts one to two years, during which all previous data is validated by the FDA [11; 14]. The NDA is a formal request for the FDA to evaluate the safety of a drug and approve it for sale in the United States [22]. According to the Office of Federal Register, the application fee in 2019 for an NDA that requires clinical data is $2,588,478 [24]. After submission of an NDA, the FDA has 60 days to file the application for review. Accordingly, the FDA reviews and acts on at least 90% of NDAs within 10 months for standard drugs, and within 6 months for priority drugs [22].

If the NDA is approved, the drug can go to market and potentially undergo a phase IV trial to evaluate long-term safety and side effects in the general public to whom the drug has been prescribed [23]. Because phase IV research is conducted over a long period on large populations, long-term and unique effects can be identified and tabulated. For example, phase IV studies are more likely to detect adverse reactions because of the larger population and presence of comorbidities [22, 25]. (See Table 1 for a summary of FDA clinical trials as well as the estimated time for each phase).

## 3. Clinical Presentation of Alzheimer's Disease

AD is a progressive disorder in which the sequelae of pathology occurring within the brain may start decades before memory loss and other cognitive symptoms appear. This stage is referred to as preclinical AD, and patients typically are symptom-free in this stage though pathological changes are taking place in the brain, particularly in the medial temporal lobe memory circuit. This network, which includes the hippocampal formation, suffers significant atrophy in AD brains. Additionally, this circuit shows reduced activity during recall tasks in AD patients [26]. The early disease process also effects the default mode network executive function circuit. This circuit includes the posterior cingulate cortex (PCC) and shows reduced activity in early AD patients compared to healthy participants [27]. The severe and early effects of AD on these regions is held in contrast to the relative protection seen in other regions, such as the cerebellum, which appears to be somewhat spared through the disease process [28]. Diagnosis of the preclinical stage and pharmacological treatment of this phase are essential to slowing the progression of the disease [29]. The preclinical phase is followed by three main stages of symptoms: mild, moderate, and severe, each characterized by an increasing severity of cognitive impairment. Thus, in the mild, early stage, symptoms include namely memory loss and problems with concentration. In the moderate, middle stage, which represents the longest stage, symptoms may include trouble remembering events, difficulty engaging in successful problem-solving thought and action, impulsive behavior, shortened attention span, language difficulties, and potential restlessness and/or agitation. In the severe, late stage, patients cannot communicate and are completely reliant on others for their care. AD is inexorably progressive and fatal within 5 to 10 years [30]. Recently, a new earlier stage of AD has been proposed and has been designated the prodromal period of AD. This transition stage of AD is also referred to as mild cognitive impairment (MCI) where the symptoms include evidence of episodic memory loss, delayed recall, decrease in executive abilities, and behavior issues most notably depression, anxiety, and sleep disturbances [31]. Interesting, with regard to depression, studies have indicated a 20-25% of AD patients suffer from a major depressive episode and another 20-30% of patients experience symptoms of minor depression [32].

## 4. The Pathology of AD: Neurofibrillary Tangles and A*β* Plaques

### 4.1. Neurofibrillary Tangles

Neurofibrillary tangles (NFTs) are intracellular lesions that represent an essential hallmark feature characteristic to AD pathology [33, 34]. NFTs are protein aggregates that show marked cellular toxicity and through this toxicity contribute to cell-wide signaling dysfunction and neuronal death [33–35]. In addition to the cytoskeletal effects of tau pathology, there is potential toxicity associated with posttranslationally modified tau and the aggregates it forms. When hyperphosphorylated tau detaches from the microtubule, it arranges into fibrous structures referred to as paired helical filaments (PHFs) [34, 36]. These fibrous and insoluble PHFs are thought to be the main components of NFTs [34, 37, 38]. Phosphorylated tau, as well as the aggregates it produces, contributes to disruptions of cell signaling and axonal transport [34, 39].

Though the toxic effects of NFTs appear to be sufficient to cause localized neuronal death, the true pathogenicity of NFTs and hyperphosphorylated tau lies in a propensity for “prion-like” spread of tangle pathology throughout the brain [40–42]. Postmortem tissue samples of AD brains show a direct correlation between confluence and location of tangle pathology with symptom progression and severity reported prior to death [40, 42]. Due to this correlation, it is believed possible that the spread of NFTs is responsible for symptom progression and characteristic Braak staging seen in AD [40, 42]. Additionally, findings of soluble tau in brain regions that, at the time of death, showed no NFTs, particularly as would have correlated with linearly progressive Braak staging had the patient lived, suggest that tau (and not the tangles themselves) is the mobile feature that allows for the spread of NFT pathology through the brain [43]. Finally, immunohistological studies of primary neurons in culture have shown that the presence of aggregated tau is sufficient to induce further misfolding of tau and therefore supports a “prion-like” method of tau and NFT spread through the AD brain [42].

### 4.2. A*β* Pathology

**Figure 2:**
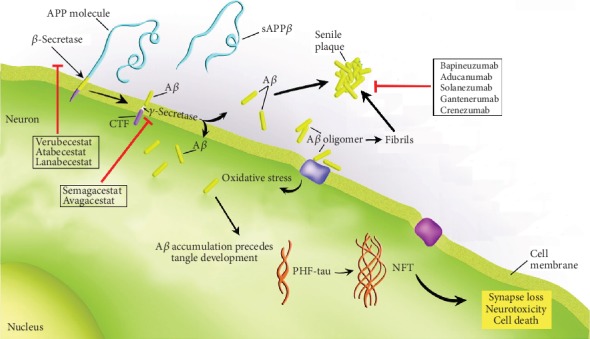
The A*β* cascade and potential therapeutics that have recently failed in human clinical trials. A highly schematic drawing illustrating the main steps involved in A*β* production and deposition as thought to occur in the AD brain. Production begins following cleavage of APP by *β*- or *γ*-secretases followed by the formation of oligomers and fibrils that eventually deposit into extracellular plaques. Broadly, therapeutics shown are targeted to either A*β* formation or clearance mediated by microglia. In addition, the connection of A*β* to eventual synapse loss and cell death is linked through tangle development.

Senile plaques are extracellular lesions characteristic of AD. These plaques are largely composed of A*β* protein aggregates that owe a significant portion of their longevity, stability, and consequent toxicity, to a beta-sheet secondary structure that proves difficult to degrade through normal microglial clearance processes [44, 45]. The A*β* protein fragment is a product of enzymatic secretase cleavage of the amyloid precursor protein (APP) [46, 47]. The proteolytic enzymes responsible for the cleavage of the precursor protein include *α*-, *β*-, and *γ*-secretase. The *α*-secretase is responsible for the “good pathway,” in which the A*β* protein fragment itself is cleaved at an extracellular scission site [48, 49]. This cleavage within the A*β* protein fragment prevents subsequent *γ*-cleavage from producing an intact A*β* peptide and thus prevents neurotoxic aggregation [46, 47, 49]. The amyloidogenic pathway requires successive cooperativity of both the *β*- and *γ*-secretases to produce an intact A*β* fragment fully freed from the cytoplasmic carboxyl terminus, as depicted in Figure 2 [47, 50, 51]. Following enzymatic cleavage by both *β*- and *γ*-secretase, lipid soluble A*β*_1-40/42_ may exert neurotoxic effects in either oligomeric or fibrillar forms [52]. In AD brains, a balanced regulation of A*β*_1-40_ and A*β*_1-42_ is disrupted such that the majority of secreted A*β* is the fibril-prone 1-42 fragment [52, 53]. This is in contrast to A*β* secretion in normally aged brains, in which 90% of A*β* is the highly soluble 1-40 fragment which adopts a fibrillar form at rates slow enough to allow for moderate clearance of the protein [52–54].

## 5. Clinical Trials Involving Disease-Modifying Therapies Targeting A*β*

### 5.1. The A*β* Hypothesis

For more than 25 years, the A*β* hypothesis (or amyloid cascade hypothesis) has been a central theory in the field of AD, positing that A*β* is the primary cause of AD, which promotes tau aggregation into NFTs, ultimately triggering neuronal death [55]. The assumption is that when A*β* aggregates (into fibrils or more specifically into oligomers), it triggers neurodegenerative processes that lead to the loss of memory and cognitive ability in AD. Strong support of this hypothesis comes from the knowledge that all known mutations that lead to early-onset AD have the overall effect of increasing the levels of A*β* [56]. Therefore, if the progression of AD is believed to align with the A*β* hypothesis, it follows that pharmaceutical intervention aimed at any discrete step along the hypothesis with regards to oligomer or plaque formation or persistence should function to retard both A*β* load and cognitive decline. This framework provides a logical backbone for the development of pharmaceuticals aiming at targeting the pathogenic mechanisms of AD, as opposed to the consequent cognitive symptoms characteristic to the dementia. These pharmaceutical approaches include those targeting the initial pathogenic processing of APP by *β*- and *γ*-secretase, as well as those concerned with the products of this enzymatic cleavage (see Figure 2).

### 5.2. Methods

The following review of clinical trial data aims at being inclusive and well-rounded, but not an exhaustive list of all previous clinical trials involving AD. The very nature of the A*β* hypothesis and its logically resultant enzymatic and immunological therapies allows for investigation into any and every discoverable discrete target that may affect A*β*. The depth of this field has therefore led to a vast capacity for redundancy, such that a large majority of related but technically different pharmaceuticals result in similar clinical disappointment, with unique findings serving as the exception to the idiomatic rule. To combat this propensity for redundancy and to maintain the efficacy of this review as a whole, the trials cited were chosen for their ability to serve as representative publications within their discrete groups. Recency of publication was not used as a strict guideline in database searches; however, a majority of chosen citations were within 15 years old. This may be due to the pace of clinical trials in general, neurologically focused clinical trials specifically, or to the relative novelty of the A*β* hypothesis in the entire scheme of AD clinical intervention.

### 5.3. Clinical Trial Results

Enzymatic cleavage of APP to A*β* relies on sequential processing of the precursor protein by the enzymes *β*-site APP cleaving enzyme 1 (BACE-1) and *γ*-secretase [49, 50]. As the release of the A*β* protein fragment from APP requires cleavage at both the *β*- and *γ*-sites, the inhibition of either site-specific enzyme is sufficient to prevent accumulation of A*β* in the extracellular space [47, 48, 50]. Drug candidates developed in this scheme include inhibitors of BACE-1 and *γ*-secretase. Evidence as to the pharmacologic engagement of BACE-1 inhibitors is often inferred from a reduction in A*β* concentrations subsequent to treatment with such an inhibitor, as determined by amyloid positron-emission tomography (amyloid PET) or immunoassay analysis [57, 58]. A 2017 study conducted by Villarreal et al., using a murine model of advanced age, showed reduction of plasma A*β*_1-40_ by 98% and A*β*_1-42_ by 90% upon treatment with BACE-1 inhibitor verubecestat. Additionally, this study found reduced levels of A*β*_1-40_ and A*β*_1-42_ (62% and 68% reduction, respectively) in cerebrospinal fluid (CSF) [59]. Human studies have shown similar results concerning the reduction of A*β* levels in response to treatment with BACE-1 inhibitor atabecestat. Results of a placebo-controlled phase I study in patients with early AD showed an up to 95% reduction of toxic A*β* levels in the CSF upon treatment with atabecestat [58]. With these promising results of reduced A*β* levels, severe ascription to the A*β* hypothesis would logically conclude parallel reductions in cognitive impairment. However, results of multicenter phase III studies of verubecestat in mild-moderate AD showed worsened cognition (similar to that observed in the placebo condition) as defined by the Alzheimer's Disease Assessment Scale-cognitive (ADAS-cog) and the Alzheimer's Disease Cooperative Study Activities of Daily Living inventory scale (ADCS-ADL) in low- and high-dose treatment conditions (12 mg/day vs. 40 mg/day) [57]. Additionally, both low- and high-dose treatment groups showed statistically significant incidences of severe adverse effects as compared to the placebo group, including elevated liver enzymes [57]. Moreover, studies of verubecestat in prodromal AD showed worsened cognition as defined by the Clinical Dementia Rating Scale-Sum of Boxes (CDR-SB) in high-dose treatment groups (40 mg/day) compared to placebo conditions, as well as continued increased incidences of adverse effects in the treatment groups [60]. Preliminary ad-hoc statistical analysis of phase II-III atabecestat studies show similar incidences of adverse events, as well as declines in measures of cognition unique from ongoing decline observed in placebo groups [61]. More recently, the BACE-1 inhibitor, lanabecestat, was abandoned by sponsors when an independent assessment of a phase III clinical trial indicated that the drug was unlikely to meet the primary endpoints [62]. Finally, adding to these failures was the recent disclosure by Novartis that it was discontinuing the investigation of the BACE-1 inhibitor, umibecestat, in two phase II-III studies due to worsening in some measures of cognitive function during a preplanned interim analysis. The sponsors concluded that the potential benefit for participants in the studies did not outweigh the risk [63].

In addition to clinical attempts at inhibition of rate-limiting *β*-secretase cleavage of APP, *γ*-secretase is also looked at as a potential target for both the reduction of A*β* and rescue of cognitive function. Similar to trials of BACE-1 inhibition, target engagement is inferred from a reduction of A*β* levels. A 2009 study of *γ*-secretase inhibitor semagacestat found a dose-dependent reduction of A*β* production up to 84% upon oral dosing of 240 mg [64]. Despite promising reductions of A*β* levels, a subsequent 2013 phase III trial found a worsened cognitive functioning as assessed by the ADAS-cog and ADCS-ADL, upon high-dose treatment as compared to placebo [65]. Additionally, significant adverse events, including increased incidence of nonmelanoma skin cancer, weight loss, and syncope, were found to be associated with treatment. These adverse effects associated with high-dose treatment (140 mg/day) resulted in higher levels of treatment discontinuation compared to the placebo (30% vs. 11%). Moreover, fatality rates were found to be higher in the treatment group receiving 140 mg compared to the placebo group (14 vs. 9 patients) [65]. Avagacestat, an additional *γ*-secretase inhibitor, was found to result in less significant reduction of A*β*_1-40_ and A*β*_1-42_ levels (10-15% and 5-9%, respectively) compared to the previously discussed drug candidates [66]. Additionally, avagacestat was shown to increase brain atrophy as measured by volumetric magnetic resonance imaging (MRI) significantly when compared to placebo [66]. Phase II studies of avagacestat showed incidences of serious adverse events at high doses (125 mg/day), similar to those observed in other *γ*-secretase inhibitors, as well as unfavorable cognitive changes as measured by ADAS-cog in high-dose treatments (100 mg/day and 125 mg/day) compared to placebo [67]. However, it does not appear that the decline in cognitive function observed in the phase II study was correlated with the observed increase in brain atrophy [67].

In addition to inhibition of the enzymes responsible for the amyloidogenic processing of APP, pharmaceutical attempts have been made to directly target the products of *β*- and *γ*-secretase cleavage. This class of A*β*-targeting drugs is typically monoclonal antibodies to A*β* in various states of solubility and aggregation [68]. For all drugs of this type, target engagement is assumed to result in either increased clearance or reduced deposition of A*β* and thus can be assessed through A*β*-specific PET analysis and immunoassay. Adherence to the A*β* hypothesis would expect decreases in A*β* concentrations to be mirrored by reductions in cognitive decline.

Antibodies specific to A*β* prior to the generation of fibrillar forms include aducanumab, solanezumab, and crenezumab. These antibodies target oligomeric, soluble, and protofibrillar A*β*. The antibody aducanumab showed moderate success in various substages of phase I trials [68]. This success included statistically significant stabilization of cognitive decline according to the Mini-Mental State Examination (MMSE) [68]. This trial was originally ended in phase III for futility, meaning that the observed effect on cognition was not replicated to statistical power in a larger trial population [69]. The interpretation of these phase III findings is held in contrast to initial studies of aducanumab which confirmed the ability of the antibody to reduce the deposition of A*β* plaques [68]. This discrepancy between apparent target engagement and clinical results may, as noted previously, indicate that A*β* is an ineffective target. However, as of October 2019, Biogen has announced that a reevaluation of this futility analysis revealed 23% less cognitive decline in a high-dose treatment group of aducanumab (10 mg/kg) compared to placebo as assessed by CDR-SB [70, 71]. This reassessment was made in response to the availability of additional data not used in the original futility analysis, and changes in outcomes were seen only in the high-dose treatment group with no additional benefit seen with other dosing, or as assessed in other subgroups of the trial [70–72]. This stark outcome discrepancy between subgroup reanalysis has sparked some doubt as to the validity of this reevaluated futility analysis in Biogen's return to seek regulatory FDA approval for aducanumab [73].

Results of phase III studies of solanezumab, an antibody targeting soluble A*β* peptides, were not able to show a significant decrease in cognitive decline when assessed by MMSE and ADAS-cog in treatment groups compared to the placebo [74]. Incidences of adverse events, including vitamin D deficiency and spinal osteoarthritis, did not differ significantly between the treatment and placebo groups (84.5% vs. 83.4%) [74]. In addition to discretely targeting soluble and insoluble forms of A*β*, it is possible to target the transition between the two, with the administration of crenezumab. Results of a phase II trial for crenezumab showed no significant stabilization or slowing of cognitive decline as assessed by the ADAS-cog or CDR-SB at either a low dose (300 mg/day) or high dose (15 mg/kg) [75]. Incidences of adverse events were found to be similar among low dose, high dose, and placebo conditions and are thus unlikely related to the administration of the drug [75]. Despite this apparent lack of effect, the high-dose treatment group was found to have significantly increased levels of CSF A*β*_1-42_. This increase in A*β* is difficult to interpret, as it may suggest either the reduced clearance of A*β* (unwanted effect) or increased mobilization of A*β* in response to reduced deposition (desired effect). A post hoc statistical analysis of this phase II trial suggests that there may be cognitive benefits derived from high-dose treatment with crenezumab as assessed by ADAS-cog (results not seen in reassessment of CDR-SB). This may suggest increased efficacy contingent on earlier and higher dose treatment with crenezumab [75].

Pharmaceutical intervention aimed at the fibrillar forms of A*β* includes the drugs bapineuzumab and gantenerumab. Results of a phase I trial of bapineuzumab show an increase in plasma A*β* levels upon high-dose treatment (5 mg/kg) [76]. This may be due to the reduced clearance of A*β*, or increased mobilization as a result of decreased deposition. Despite this uncertainty, phase III results, confirming the reduction of fibrillar A*β* in response to treatment with bapineuzumab, would suggest increased mobilization of A*β* as the mechanistic cause of the observed transient increase in A*β* levels [77]. However, despite the confirmed reduction of fibrillar A*β* levels, results of phase III trials showed no significant differences in cognitive decline between placebo and treatment groups as assessed by the ADAS-cog and the Disability Assessment for Dementia (DAD) [78]. Significant adverse events recorded in response to bapineuzumab include the presence of asymptomatic amyloid-related imaging abnormalities [78]. Finally, phase III studies of fibril targeting gantenerumab were stopped early for futility due to a lack of statistical significance between treatment (high dose: 225 mg; low dose: 105 mg) and placebo groups in measures of brain atrophy (as measured by volumetric MRI), CSF A*β* concentrations, cognitive decline (as measured by CDR-SB, MMSE, and ADAS-cog), and incidences of treatment or disease related adverse events [79].  Table 2 summarizes findings from recent clinical trials targeting the beta-amyloid cascade.

Though the A*β* hypothesis lends itself well to the enzymatic and immunological therapies previously discussed, these strategies do not describe all A*β*-focused treatment. The Alzheimer's Management By Albumin Replacement (AMBAR) study is an ongoing multicenter trial currently in phases II-III. AMBAR involves plasma exchange treatment with albumin replacement in an effort to increase A*β* mobilization. The primary trial outcome is cognitive change as assessed by the ADAS-cog and ADCS-ADL [80].

A brief discussion of the current tau-targeted trials is warranted, as more focus than ever begins to be placed on targets other than A*β*. Similar to the immunotherapeutic strategies attempting to target A*β*, many current trial attempts focus on passive immunization to both pathological and native forms of tau [81, 82]. C2N 8E12 is a passive immunotherapeutic approach to targeting tau. This antibody is specific to aggregated tau as it exists extracellularly in NFT pathology. Phase I of this trial focused not on AD but on supranuclear palsy and found that the drug itself was safe at dosages from 2.5 to 50 mg/kg. This trial is currently in phase II with randomized dosage to determine efficacy [82]. RO7105705, or semorinemab, is another passive immunization against extracellular tau and targets a phosphorylated serine residue of pathological tau. Phase I of this trial found no serious adverse events related to drug administration and a bioavailability of 70% when administered subcutaneously [83, 84]. This trial is currently in phase II and plans to use the ADAS-cog and ADCS-ADL as the primary endpoints to measure cognitive change over the course of treatment [81, 83, 84].

### 5.4. Potential Causes of Trial Failure

Nearly consistent trial failures cast significant doubt on the validity of the A*β* hypothesis. However, there may be issues with the methods commonly used in AD clinical trials that complicate attempts to appropriately analyze results of these trials. These issues should be considered and addressed in moderate isolation from the growing concerns about hypothesis validity. For example, potential weaknesses of trial design begin with the designation of clinically meaningful endpoints identified by the protocols put forth in the IND. Achievement of endpoints signifies success of the trial and presumes replicable results in a large consumer-patient population. In AD trials, the most clinically significant (direct) endpoints are cognitive improvement and rescued brain activity [85]. In addition to direct endpoints, the meeting of indirect endpoints such as changes to biomarkers may also be used in the trial process due to the relative ease and reduced cost of these assessments. In AD trials, the biomarkers in question are CSF concentrations of A*β*_42_, total tau, and p-tau. Automated immunoassays (such as ELISA) available to quantify concentrations of these biomarkers can be useful in the diagnosis of AD, even at very early stages [86–88]. However, despite usefulness as a diagnostic tool, reliance on these biomarkers (and changes in biomarker characteristic over the course of a clinical trial) as endpoints is not sufficient [85]. This is seen to be the case in the previously discussed trials of verubecestat, atabecestat, and semagacestat [58, 61, 64]. Trials of these drugs show promising findings of reduced A*β* concentrations with worsened or unchanged cognition. In addition to immunoassays, A*β*-specific PET scans can be conducted to assess changes in concentration of this protein. Unfortunately, this measure falls prey to the same issues as seen with immunoassays in that decreases of a target *presumed* to be a causative peptide in the disease process, though promising, mean very little if not mirrored by cognitive improvement. In contrast to these subjective endpoints, PET scans can be used to show changes in metabolic activity as a result of trial intervention [89, 90]. Specifically, PET scans would look for increased activity in the posterior cingulate and temporoparietal regions, which show significantly lessened metabolism in the AD brain when compared to a healthy control [89, 90]. Additionally, the use of biomarker reduction as an indicator of target engagement, though superficially logical, is particularly inadequate when concerning A*β*-targeting immunotherapies, due to the potential for antibody cross-reactivity (though cross-reactivity is much less likely in clinically useful monoclonal strategies than other antibody-based therapeutics) [91, 92]. In other words, the reduction of toxic A*β* in response to treatment with a monoclonal antibody does not summarily imply the binding of such antibody to A*β* itself or to the correct epitope [91–93]. The antibody may in fact be binding to an unintended target that, due to a secondary mechanism, results in the reduction of A*β*. However, any unintended binding may have additional side effects that complicate trial results [91, 93].

Rescue or stabilization of cognition is the ultimate aim of all AD trials. As such, cognitive testing is often used as a direct endpoint to assess the efficacy of the drug as it relates to this overarching goal. However, the administration of these tests is subjective and can vary significantly between testers, as well as between trial sites. To point, a 2008 study of the ADAS-cog showed that variation in tester procedure resulted in significantly unreliable scoring [94]. Additionally, a 2015 study of the ADAS-cog showed low reliability for measurement of cognitive change between administrations of testing in the same patient, though reliability for discrete testing was adequate [95]. If this lack of reliability for measuring change in cognition is correct, then any study which uses ADAS-cog (including the previously discussed trials of verubecestat, semagacestat, avagacestat, solanezumab, crenezumab, and bapineuzumab) in this manner may not be reporting accurate findings on cognitive impairment, the most important aim of clinical AD intervention.

A final reason clinical trials in AD may be not reaching predesigned endpoints is the analysis of the placebo-controlled group especially if the sample size is insufficient. For example, unusual improvement in the placebo group limits the ability to determine if the drug is indeed effective. On the other hand, a rapid decline in the placebo group may be misleading in suggesting an overly strong benefit from the therapeutic. This in turn might falsely convince sponsors to under-power a subsequent trial expecting a similarly robust effect [96].

## 6. Future Directions

As the plight of AD grows in magnitude and cost unmet by definitively effective A*β*-based treatment, more emphasis is being placed on the discovery of new targets and formulation of accurate hypotheses of disease progression. Additionally, trial failures provoke introspection into the validity of methods and procedures. Beginning with the use of animal models to predict efficacy and toxicity in preclinical research, there may be room for improvement. Though animal models provide undeniably important data in research, these studies are able to predict human toxicity correctly in only 70% of cases [97, 98]. Because these animal toxicity studies are done early in the trial process, this may allow for a significant proportion of drugs with potential toxicity in humans to unduly continue in the trial process. Take for instance the trial results of the initial BACE-1 inhibitor studies, in which liver toxicity was not noted in preliminary mouse models despite being a driving force in the termination of the trials [57, 99]. This is not to say that the complete removal of animal studies in the clinical trial process is warranted or prudent, only that caution needs to be exerted when extrapolating results from mouse models to humans, particularly in AD research. One methodology that might help mitigate the impact of relying on animal models is the use of induced pluripotent stem cells (iPSC) derived from humans with AD. The use of these cells would allow for investigation into tissue- and cell-specific toxicity of human cells and, more importantly, human cells with potential unknown modifications that are found in AD [100]. Additionally, the cost of generating and validating a research-grade cell line of iPSCs ranges from $10,000 to 25,000, presenting a nominal price increase in the entire scheme of drug manufacturing [101]. However, no method is without fault, and the use of iPSCs finds its flaw in a potential lack of ability to accurately phenocopy the behavior of a pharmaceutical in a complex 3-dimensional structure such as is found in the brain with over 100 billion neurons and trillions of synapses, as opposed to the 2-dimensional structures found in cell culture [102, 103].

Future attempts at targeted AD treatment may involve the use of antisense oligonucleotides (ASO) to alter target expression. These strategies may be especially useful against tau pathology, as murine models have already shown improved learning and memory in shock avoidance tasks in response to ASO treatment against GSK-3*β*, one of the primary kinases responsible in the hyperphosphorylation and subsequent dysfunction of tau [104]. Additionally, this same study showed decreased markers of oxidative stress, typically found in tissues affected by tau pathology, in response to treatment with RNA interference [104]. However, despite these promising results, the use of ASOs in clinical treatment of neurodegenerative disorders has historically been expensive to the point of inaccessibility, even for patients with health insurance [105, 106]. Additionally, ASOs find limitations in their ability, or relative lack thereof, to cross the BBB and interact with the appropriate targets [107].

In the face of A*β* hypothesis-driven clinical failure, even given the potential success of aducanumab, there is increasing interest in the discovery of new biomolecular targets for AD treatment. Both proteomic and transcriptomic approaches may be used to identify new potential targets, as well as to examine the effects of specific treatments on these targets [108, 109].

## 7. Conclusions

Alzheimer's disease (AD) is a progressive and fatal neurodegenerative disorder that primarily affects older adults and is the most common cause of dementia [110]. Currently, it afflicts 5.5 million Americans, and that number is expected to triple by 2050. To date, AD is the third leading cause of death behind heart disease and cancer, with 700,000 Americans age > 65 years estimated to suffer from AD when they die [111]. In addition, the cost of the disease is substantial with $259 billion healthcare dollars going to manage the disease currently, and by the middle of the century, costs are predicted to soar over $1.2 trillion, which will completely bankrupt the healthcare system in the USA [112]. It is clear from these data that effective disease-modifying medications are urgently needed for patients. Current FDA-approved medications including cholinesterase inhibitors and NMDA agonists provide symptomatic relief and have low efficacy. Indeed, a study of cholinesterase inhibitors and NMDA recently concluded that these drugs have weak beneficial effects on cognitive function [113]. In addition, a significant investment into clinical trials targeting different aspects of the A*β* hypothesis has proven futile up to this point. To date, the pharmaceutical industry has spent over $3.5 billion in Alzheimer's research and development in the last 4 years with a 99.6% failure rate [96]. It is often said we learn more from our failures than successes and if that is indeed true, we should accept that A*β* may be an effect not a cause of dementia. Therefore, it is our opinion that the Alzheimer's field should continue to examine A*β* strategies (particularly in light of Biogen's preemptive submission to the FDA for aducanumab's approval) but also focus on other potential drug targets including but not limited to pathological forms of tau, antisense oligonucleotides (ASOs), or other genetic approaches including CRISPR. In addition, other potential targets that are seeing early success in clinical trials include those targeting telomerase, MAOB, nuclear receptors, neurogenesis, and p38a inhibitors [114]. Clinical efforts should also double down on pathological forms of tau particularly in light of two recent articles suggesting that tau far surpasses amyloid in predicting the location of future brain atrophy. In the first study, beta-amyloid plaques appear to be latecomers to the disease rather than an early trigger in that no statistical difference in plaque load was found during the earliest, subtle cognitive difficulties, suggesting that beta-amyloid may not be the trigger for initial disease progression [115]. In the second study, using tau PET imaging (currently under review by the FDA), the authors eloquently demonstrated in early clinical stage AD patients, tau PET brain scans predict the location of brain atrophy measured by MRIs 1-2 years later, but amyloid PET imaging neither predicts the location of either tau nor future atrophy [116].

Finally, preclinically, previous results have shown that AD animal models do not predict human efficacy or toxicity; therefore, future approaches should expand to include the use of induced pluripotent stem cells derived from humans with AD in addition to the continued use of appropriate animal models. The use of these cells would predictably allow for a better recapitulation of the human AD disease process that may translate more favorably in terms of drug toxicity and efficacy. New therapies that prevent, slow, or stop the disease are urgently needed to fight the growing Alzheimer's disease burden in the United States and around the world.

## Figures and Tables

**Table 1:** Clinical trial phases as regulated by the FDA. Table detailing FDA-regulated clinical trial phases as a function of their length, purpose, and test population. ^∗^IND is not a distinct phase of the clinical trial process but is a required application between the preclinical phase and phases I-IV.

	Length of phase (years)	Purpose	Test population
Preclinical	6.5	Toxicity studies	Nonhuman animals
IND^∗^	N/A	Successful completion of phase I Allows for clinical trial to be commenced	N/A
Phase 0	7 days	To show whether a drug's pharmacokinetics and pharmacodynamics warrant continued exploration	10-15 healthy volunteers
Phase I	1	Toxicity Pharmacokinetics	20-80 healthy volunteers
Phase II	2	Pharmacokinetics Efficacy Major side effects	100-300 patient volunteers
Phase III	3	Safety Efficacy	1,000-3,000 patient volunteers
FDA	1-2	Reviews NDA Validates data	N/A
Phase IV	N/A	Postmarketing surveillance trial	Public, entire population (prescribed treatment)

**Table 2:** Recent clinical trial failures involving the beta-amyloid hypothesis.

Agent	Target/mechanism [relevant references]	Trial phase	Reasons for failure	Comments
	Gamma secretase inhibitors			
Semagacestat	[65]	III	Toxicity and lack of efficacy	Worsens cognition
Avagacestat	[66]	II	Toxicity and lack of efficacy	

	Monoclonal antibodies to A*β* or its oligomers or fibrils			
Bapineuzumab	[78]	III	Lack of efficacy	Asymptomatic amyloid-related imaging A*β* normalities
Aducanumab	[68, 69]	III	Futility analysis	Prediction that trials would not improve cognition
Solanezumab	[74]	III	Lack of efficacy	Also tested on prodromal AD
Gantenerumab	[79]	II	Lack of efficacy	
Crenezumab	[75]	II	Lack of efficacy	

	BACE-1 inhibitors			
Verubecestat	[57]	III	Lack of efficacy	Worsens cognition
	[60]	III	Lack of efficacy	Tested on prodromal AD
Atabecestat	[61]	III	Toxicity	Worsens cognition
Lanabecestat	[62]	III	Lack of efficacy	Worsens cognition
